# Fahr’s Syndrome: A Rare Case Presentation

**DOI:** 10.7759/cureus.47812

**Published:** 2023-10-27

**Authors:** Muhammad Hayyan Wazir, Yamna Ali, Ahmad Z Mufti, Alvina Ahmad, Hasnain Ahmad

**Affiliations:** 1 Internal Medicine, Hayatabad Medical Complex Peshawar, Peshawar, PAK

**Keywords:** computed tomography (ct ), seizure disorders, calcium deposits, hypoparathyriodism, basal ganglia

## Abstract

Idiopathic basal ganglia calcification (IBGC), also known as Fahr's disease, is a rare neurological disorder characterized by metabolic, biochemical, neuroradiological, and neuropsychiatric alterations resulting from symmetrical and bilateral intracranial calcifications. In most cases, an autosomal dominant pattern of inheritance and genetic heterogeneity is observed. Neuropsychiatric symptoms with movement disorders account for 55% of the manifestations of this disease.

In this report, we present the case of a 42-year-old Pakistani male who presented to the emergency department with a sudden onset of generalized tonic muscle contractions. His medical history revealed progressive cognitive impairment, and he had a history of taking oral calcium supplements. Initial laboratory investigations showed hypocalcemia with normal magnesium and phosphate levels, while his parathyroid hormone levels were low. The interictal electroencephalogram was normal, and CT imaging of the brain without contrast revealed bilateral symmetrical dense calcifications in the basal ganglia, thalami, periventricular area, corona radiata, centrum semiovale, and dentate nucleus of the cerebellum, suggestive of Fahr disease. Intravenous calcium gluconate was administered in the emergency department, leading to an improvement in the patient's symptoms. The diagnosis of IBGC with relevant symptoms was confirmed through laboratory values and characteristic features observed in the CT examination.

## Introduction

Fahr's disease, a rare genetic disorder, is a neurodegenerative abnormality of calcium deposition in the brain parenchyma. It is a rare disorder with a prevalence of < 1/1,000,000 [1].

Calcium deposits in the brain are common incidental findings related to aging. However, symmetrical bilateral calcium buildup in the brain tissue, mainly in the basal ganglia, thalamus, cerebral cortex, dentate nucleus, and cerebellum, unaccompanied by underlying pathology, is termed Fahr's disease [2]. It was first reported by Karl Theodor Fahr, a German neurologist, in 1930 [1]. It is expressed in an autosomal dominant inheritance pattern, predominant in the fifth and sixth decade of life while the symptoms are most similar to parkinsonism like bradykinesia, rigidity, tremor, hypophonia, hypomimia, mask-like facies, and a shuffling gait [3].

The diagnostic criteria for Fahr's syndrome include bilateral striatopallidodentate calcification on neuroimaging, progressive cognitive dysfunction, and movement disorders without biochemical, infectious, toxic, and traumatic causes [4]. A computed tomography scan remains the best imaging modality for Fahr's disease [5].

## Case presentation

The patient was a 42-year-old Pakistani male with no previous co-morbidities who presented with sudden onset of generalized tonic muscle contractions of about four minutes duration followed by rigidity, frothing from the mouth, clumsiness and gained conscious level within 10 minutes. However, it was not accompanied by tongue bites or urinary and fecal incontinence. The patient had a history of progressive cognitive impairment and a drug history of oral calcium supplements intake prescribed by a doctor after an episode of the same symptoms three years back, but no medical records were available. He had poor compliance with calcium supplements. Family history was unremarkable for similar conditions.

On physical examination, the patient was afebrile, had a blood pressure of 110/70mmhg, a pulse rate of 86 beats per minute, oxygen saturation of 96% on room air, and random blood sugar levels of 146mg/dl. Glasgow Coma Score of the patient was 15/15, drowsy but arousable. Deep tendon reflexes were diminished. No focal sensory deficit was detected at the time of presentation. No signs of cerebellar disease were detected. rest of the examination was unremarkable.

Laboratory investigations showed low calcium levels of 4.8mg/dL (normal:8.00-10.00mg/dL), serum magnesium level of 0.88mmol/L (normal 0.66-1.033mmol/L), parathyroid hormone level of 1.1pg/mL (normal: 10.0-69.0pg/mL) and serum phosphate level of 0.98mmol/L (normal: 0.8-1.4mmol/L). The serum electrolytes were normal with sodium of 145mmol/L (normal: 135-150mmol/L), potassium of 3.9mmol/L (normal: 3.5-5.1mmol/L), and chloride level of 98.5mmol/L (normal: 96-112mmol/L). The electrocardiogram showed sinus rhythm and a normal QTc of 458ms (451-470ms). The thyroid panel was not evaluated. Lipid profile, liver function, and renal function tests were unremarkable. Levels of Vitamin D were also normal ruling out low Vitamin D as the cause of low calcium levels. The electroencephalogram report was also unremarkable.

**Figure 1:**
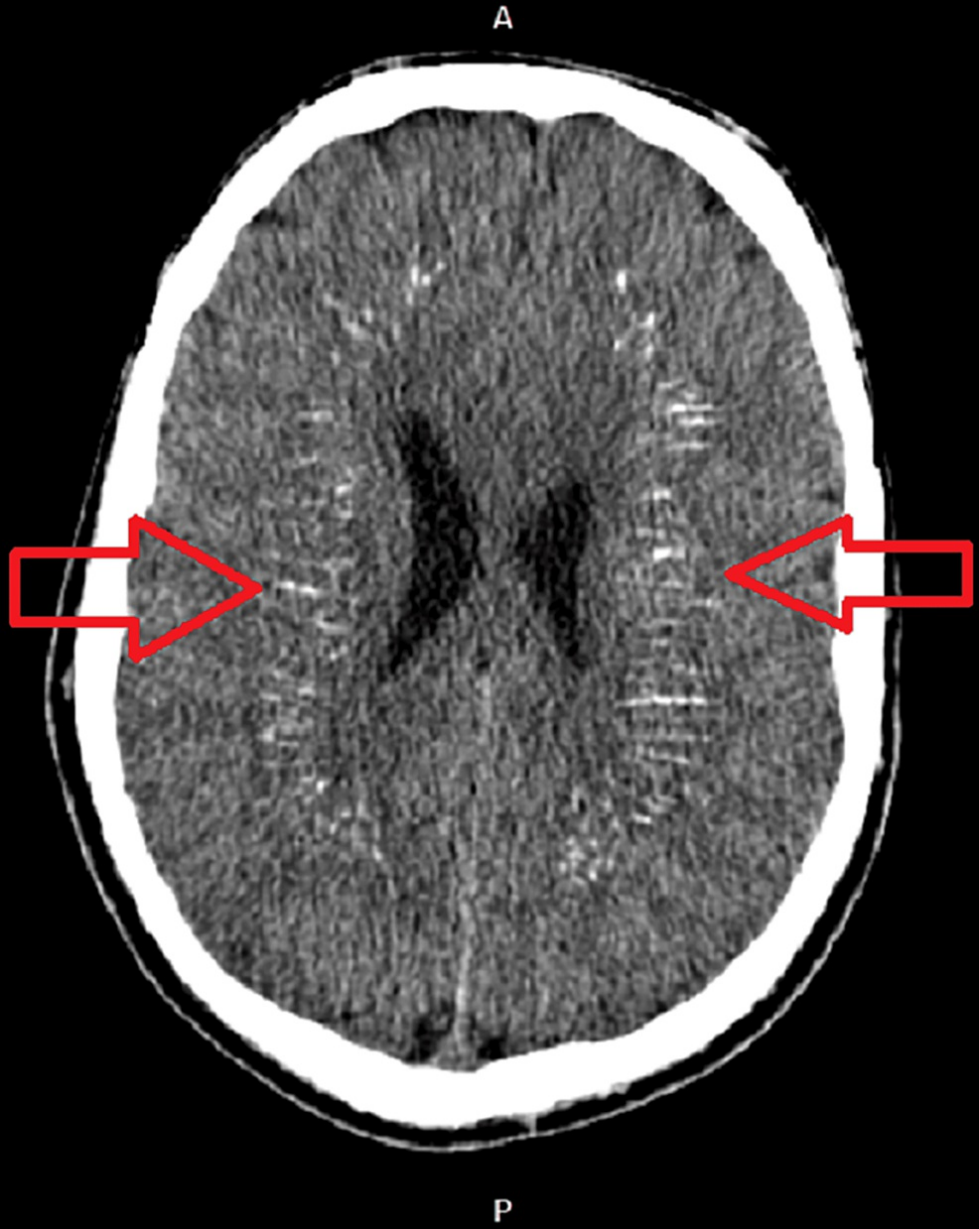
Non-contrast computed tomography image showing bilateral symmetrical calcifications in basal ganglia, thalami and periventricular area (red arrow).

**Figure 2:**
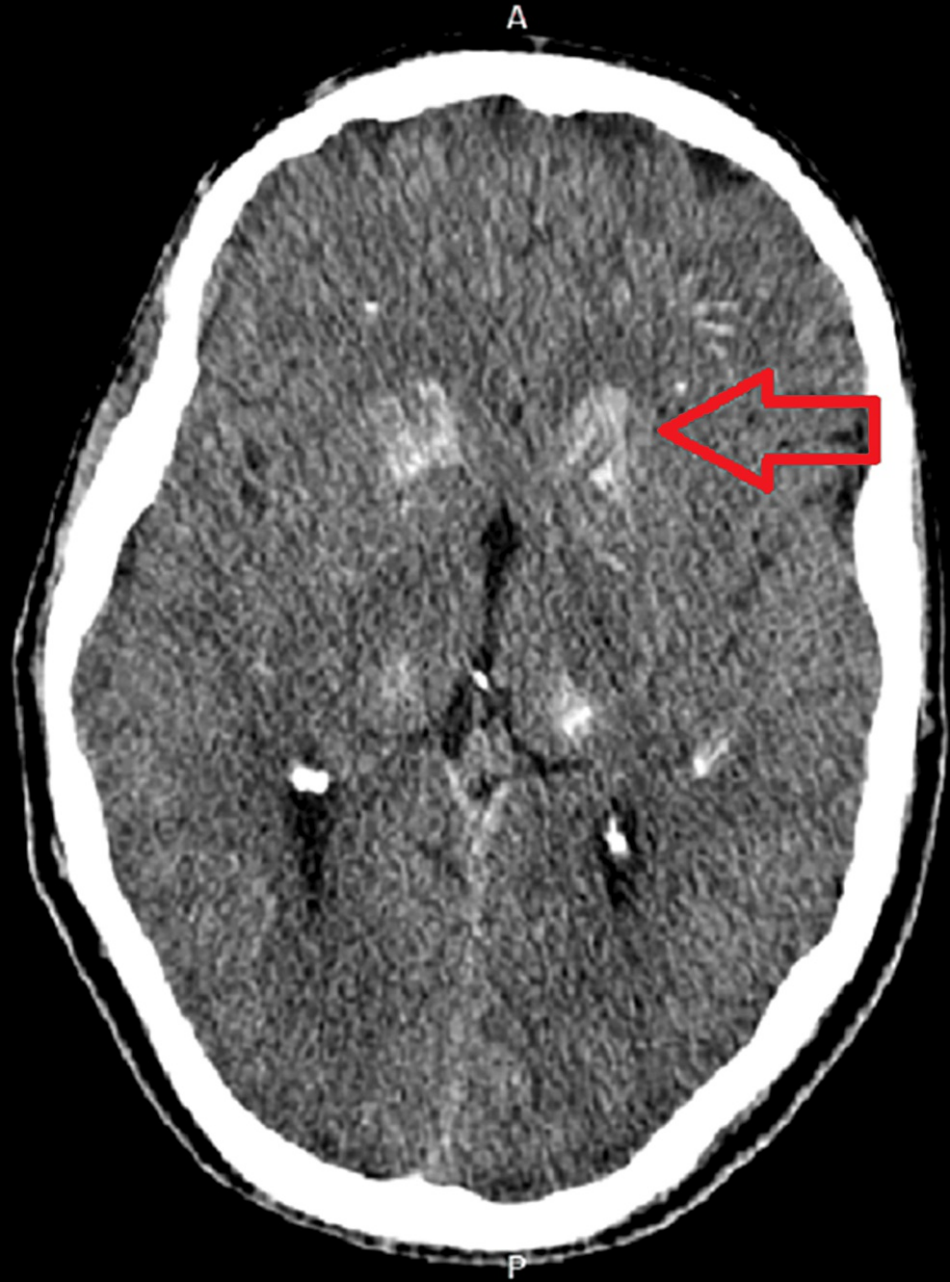
Non-contrast computed tomography image showing bilateral dense calcifications (red arrow).

CT imaging of the brain without contrast revealed bilateral symmetrical dense calcifications in basal ganglia, thalami, periventricular, corona radiate, centrum semiovale, and dentate nucleus of the Cerebellum, suggestive of Fahr syndrome (Fig 1,2). There was no evidence of intracranial bleeding or territorial ischemia. The patient was given intravenous calcium gluconate in the emergency department, and the patient’s symptoms improved. the patient was asked to come for a follow-up after two weeks and on the follow-up visit, he was doing fine.

## Discussion

Fahr’s syndrome is a disorder characterized by bilateral calcification of basal ganglia and the dentate nuclei. This intracranial calcification is usually bilateral and symmetrical, also called Bilateral Striopallido Dentate Calcinosis (BSPDC) or Idiopathic Basal ganglia calcification [6]. The signs and symptoms of Fahr’s syndrome include neurological manifestation (such as seizures and tetany), motor manifestation (includes lethargy, uncontrolled choreatic movement, and muscular cramps), and neuropsychiatric ailments (for instance, early onset dementia, impaired cognition, alteration in behavior and personality). This syndrome is most often associated with hypoparathyroidism. Other conditions include neuroferritinopathy, Kenny-Caffey syndrome type 1, intrauterine or perinatal infection (e.g., toxoplasma gondii, rubella), tuberous sclerosis complex, brucella infection have also been associated with Fahr’s syndrome [7]. Fahr’s disease, like Fahr’s syndrome, presents with the same clinical manifestation; however, it is a rare hereditary disease with an autosomal dominant pattern of inheritance, presentation usually seen in the fourth to sixth decade of life along with the absence of association to any trauma, infection, metabolic and mitochondrial disease or any systemic disease [8]. Mutation in specific genes such SLC20A2, PDGFRB, PDGFB, XPR1, and myogenic regulating glycosylase genes were observed in Fahr’s disease [9]. Fahr’s syndrome’s prevalence is <1/1,000,000, making it a rare neurodegenerative disorder [10].

Diagnostic workup of Fahr’s syndrome starts with recognizing clinical manifestation and identifying bilateral basal ganglia calcification on CT/MRI brain [11]. Investigation for parathyroid disorders (for primary and secondary hypoparathyroidism) and abnormalities in calcium metabolism should be done for its exclusion, as calcification of basal ganglia related to it is a common entity in adults [12]. Further investigations should be done to exclude other conditions like hypoparathyroidism, pseudohypoparathyroidism, mitochondrial myopathy, brucellosis, neurocysticercosis, toxoplasmosis, and tuberous sclerosis complex [9]. In the case of Fahr’s disease, family history will suggest that the inheritance pattern, along with molecular genetic testing, should be made to observe for any genetic mutation [1]. Lastly, physiological basal ganglia calcification should be differentiated from pathological calcification. These calcifications have been detected in up to 20% of CT scans, with their predilection involving globus pallidus, pineal gland, falx, and choroid plexus [9,13].

Management protocol for the patient affected with Fahr’s syndrome fixates on pharmacological treatment for relieving the symptoms. Hypoparathyroidism and other disorders of Calcium metabolism are managed with the use of intravenous calcium gluconate or the use of oral calcium and vitamin D supplements to alleviate seizures, extrapyramidal signs, and paresthesia [14]. Furthermore, suitable anti-epileptic and anti-parkinsonian drugs should be used in patients with seizures or parkinsonian features [14,15]. Anti-psychotic medication can be used cautiously in patients with features of psychosis, irritability, and other behavioral changes, keeping in mind the exacerbation of extrapyramidal symptoms associated with these drugs [16]. Recently, a study that revealed the use of alendronate in patients showing improvement in symptoms such as tremors and headache with better tolerability proposed the effectiveness of this treatment in Fahr’s syndrome [17].

## Conclusions

In summary, this case report sheds light on the clinical manifestations and diagnostic complexities linked to Fahr's syndrome an uncommon neurodegenerative condition marked by the bilateral calcification of basal ganglia and dentate nuclei. Our 42-year-old male patient presented with sudden-onset generalized tonic muscle contractions that significantly improved following intravenous calcium gluconate administration. Laboratory results revealed hypocalcemia, underscoring the significance of considering calcium metabolic disorders during the differential diagnosis of Fahr's syndrome. Furthermore, this case emphasizes the necessity of meticulous assessments, including neuroimaging, to distinguish pathological calcifications specific to Fahr's syndrome from incidental findings. Therapeutic interventions encompass calcium and vitamin D supplementation, anti-epileptic and anti-parkinsonian medications, and cautious administration of antipsychotic drugs to manage symptoms. This case emphasizes the rarity of Fahr's syndrome and highlights the vital role of a multidisciplinary approach in its diagnosis and therapeutic management.
